# First trimester antidepressant use and miscarriage: a population-based cohort study using Clinical Practice Research Datalink GOLD

**DOI:** 10.3399/BJGP.2025.0092

**Published:** 2025-11-18

**Authors:** Florence Z Martin, Paul C Madley-Dowd, Viktor H Ahlqvist, Gemma C Sharp, Kayleigh E Easey, Brian K Lee, Abi Merriel, Dheeraj Rai, Harriet Forbes

**Affiliations:** 1 Medical Research Council Integrative Epidemiology Unit, Bristol Medical School, University of Bristol, Bristol, UK; 2 Population Health Sciences, Bristol Medical School, University of Bristol, Bristol, UK; 3 Centre for Academic Mental Health, Bristol Medical School, University of Bristol, Bristol, UK; 4 NIHR Bristol Biomedical Research Centre, University Hospitals Bristol and Weston NHS Foundation Trust and University of Bristol, Bristol, UK; 5 Department of Biomedicine, Aarhus University, Aarhus, Denmark; 6 Institute of Environmental Medicine, Karolinska Institutet, Stockholm, Sweden; 7 School of Psychology, University of Exeter, Exeter, UK; 8 School of Psychological Science, University of Bristol, Bristol, UK; 9 AJ Drexel Autism Institute, Drexel University, Philadelphia, PA, US; 10 Department of Epidemiology and Biostatistics, Drexel University Dornsife School of Public Health, Philadelphia, PA, US; 11 Department of Global Public Health, Karolinska Institutet, Stockholm, Sweden; 12 Women's and Children's Health, Institute of Life Course and Medical Sciences, University of Liverpool, Liverpool, UK; 13 Department of Non-communicable Disease Epidemiology, London School of Hygiene and Tropical Medicine, London, UK

**Keywords:** depression, epidemiology, obstetrics and maternal health, antidepressants, cohort studies, primary health care

## Abstract

**Background:**

Depression and anxiety during pregnancy is on the rise, thus more pregnant women are being offered antidepressants; however, uncertainties remain surrounding safety.

**Aim:**

To investigate the association between first trimester antidepressant use and miscarriage.

**Design and setting:**

Population-based cohort study using the UK Clinical Practice Research Datalink (CPRD) GOLD.

**Method:**

Pregnancies included in the CPRD GOLD Pregnancy Register between 1996 and 2018 were identified. Pregnancies in those with prescriptions for antidepressants overlapping with the first trimester were defined as ‘exposed’ and compared with pregnancies in those who were unexposed. Cox models, adjusted hazard ratios (aHRs), and absolute risk of miscarriage were calculated adjusted for confounders including depression, anxiety, smoking, and other health, lifestyle, and obstetric factors.

**Results:**

Among the 1 021 384 eligible pregnancies, 73 540 patients were prescribed antidepressants in the first trimester (7.2%); 10 693/73 540 (14.5%) pregnancies ended in miscarriage among those prescribed antidepressants versus 116 641/947 844 (12.3%) in those not prescribed antidepressants. Antidepressant prescription during the first trimester was only modestly associated with miscarriage following adjustment (aHR 1.04, 95% confidence interval [CI] = 1.02 to 1.06). These findings translated to an absolute risk adjusted for confounders of 13.1% (95% CI = 13.0 to 13.2) for those not prescribed and 13.6% (95% CI = 13.3 to 13.8) for those prescribed antidepressants. Among those prescribed antidepressants in the 3 months before pregnancy and during the first trimester, the risk of miscarriage was the same as among those unexposed (aHR 1.00, 95% CI = 0.98 to 1.03).

**Conclusion:**

First trimester antidepressant use was associated with a small, clinically insignificant increased risk of miscarriage, with no evidence suggesting taking antidepressants before pregnancy and into the first trimester increases the risk of miscarriage.

## How this fits in

Antidepressant use during pregnancy is rising and evidence is conflicting as to whether these medications increase the risk of miscarriage if taken during pregnancy. This study used the Clinical Practice Research Datalink GOLD, a large repository of UK-based primary care data, and a range of methods to investigate antidepressant use during trimester one and risk of miscarriage. The study found that antidepressant use during pregnancy was associated with a small but clinically insignificant risk of miscarriage. These results, plus the estimated prevalence of miscarriage among both groups, can be used in clinical practice to support evidence-based decision making for women planning pregnancy or becoming pregnant on antidepressants with concerns around miscarriage risk.

## Introduction

Antidepressant use during pregnancy is prevalent in many countries, with estimates suggesting that upwards of 8% of pregnant people use antidepressants at some point during pregnancy.^1–3^ Although most antidepressants are not contraindicated during pregnancy, they are prescribed with some caution because of evidence suggesting small increases in risk of miscarriage, preterm delivery, and postpartum haemorrhage.^4–9^ In the UK, the National Institute for Health and Care Excellence (NICE) updated its guidance in 2023 (and again in 2025) from severity-based advice to patient-centred decision making when planning pregnancy or becoming pregnant on antidepressants, weighing up the risks to both the pregnant person and baby on an individual basis.^10–12^ Globally, the guidance around using antidepressants during pregnancy is mixed,^13–15^ reflecting the uncertainty in the evidence base and, in turn, challenges faced by prescribing clinicians and patients.^4^

The definition of miscarriage varies, but is mostly defined as a pregnancy loss before 20–24 weeks’ gestation.^16,17^ A systematic review and meta-analysis of 29 studies identified a modest increased risk of miscarriage following any antidepressant use during pregnancy (pooled odds ratio 1.24, 95% confidence interval [CI] = 1.18 to 1.31).^18^ Biologically, it is plausible that antidepressants could causally increase the risk of miscarriage, owing to their inhibition of serotonin transporters on platelets and subsequent association with bleeding events.^19^ However, untreated depression and anxiety during pregnancy are also associated with adverse pregnancy outcomes, like preterm birth and low birth weight.^20–23^ Thus, the link between antidepressant use during pregnancy and miscarriage could be explained by the underlying disease for which antidepressants are prescribed^1,24^ rather than the drugs themselves, known as confounding by indication. Given the use of general population controls in several studies^18^ and some studies omitting indication adjustment completely,^25–31^ it is not possible to conclude a causal relationship between antidepressants and miscarriage from the present literature. The dearth of estimated absolute risks reduces clinical interpretability for evidence-based decision making in primary care.

In this cohort study, Clinical Practice Research Datalink (CPRD) GOLD data were used to investigate first trimester antidepressant use and miscarriage using a range of methodological approaches, including an exposure-discordant pregnancy design, propensity-score matching, and stratified analyses, to help account for confounding by, and severity of, indication. The absolute risk adjusted for confounders was estimated to situate the estimates of relative risk in the context of the ‘baseline’ risk.

## Method

### Data sources

CPRD GOLD is a UK-based repository of anonymised general practice data and makes up part of one of the largest resources of primary care data in the world.^32^ It covers approximately 7% of the UK’s population, representative by age, sex, and ethnicty.^32^ The primary care data in CPRD GOLD are linked to practice-level Index of Multiple Deprivation (IMD) scores and Hospital Episode Statistics Admitted Patient Care for most English practices.^32^

The CPRD GOLD Pregnancy Register is described in detail elsewhere;^33^ in short, it contains pregnancy episodes with estimated pregnancy dates and outcomes linked to patient identifiers in CPRD GOLD (see Supplementary Information S1). In the current study, the Pregnancy Register was prepared in accordance with recommendations from the authors of the register algorithm.^34^

Pregnancy episodes with an uncertain outcome were recoded where Hospital Episode Statistics data were available, and dates were updated using imputed values as per the Pregnancy Register algorithm, whereby a set gestational length is used depending on the updated outcome (see Supplementary Information S2). Pregnancy episodes ending in ‘unknown outcome’ that were not recoverable using Hospital Episode Statistics were excluded.

### Study design and population

Inclusion into this cohort study was defined as the following: those with ≥1 pregnancy episode between 1996 and 2018 who were registered with an ‘up-to-standard’ practice^32^ and had adequate follow-up for at least a year before and up until the end of pregnancy, meaning they did not leave their practice, have a death date, or have a last collection date before the end of pregnancy.

### Antidepressants

All antidepressants approved for treating depression in the UK were identified and stratified by class (see Supplementary Table S1). Briefly, prescription end date was used in conjunction with the pregnancy start date and the end date of trimester one to identify whether an antidepressant prescription occurred within, or overlapped with, the first trimester to identify those ‘exposed’ to antidepressants (see Supplementary Information S3).

Dose was standardised for each medication using the distribution of dose in milligrams; low (≤25th percentile), medium, and high (>75th percentile) doses (see Supplementary Information S3). In instances where multiple doses were prescribed in trimester one, individuals were classified with the highest dose they received in the first trimester.

### Confounders

Confounders were chosen a priori based on the literature. The primary adjustment set contained: age; year of pregnancy; IMD quintile (as a proxy for practice-level socioeconomic deprivation); history of miscarriage and severe mental illness; smoking; parity; use of high-dose folic acid, antipsychotics, and antiseizure medications; number of primary care consultations in the 12 months before pregnancy; and whether an individual has ever been diagnosed with depression and anxiety before the start of pregnancy (see Supplementary Information S4, Supplementary Table S2, and Supplementary Figure S1).

Depression and anxiety were identified using pre-defined, expert-verified codelists in primary care (Read codes) and Hospital Episode Statistics Admitted Patient Care (International Classification of Diseases, 10th Revision codes).

Ethnicity^35^ and body mass index (BMI) around the start of pregnancy contained >10% missing data, thus were dropped from the primary adjustment set and included in sensitivity analysis.

### Analysis

Baseline characteristics of the eligible sample are described by first trimester antidepressant use. All analyses were performed using complete records for covariates.

First, those prescribed antidepressants in trimester one were compared with those who were not, using Cox proportional-hazards models. ‘Incident’ users (those who were not prescribed antidepressants in the 3 months before pregnancy but were in trimester one) contributed non-prescribed time to the analysis until the start of their antidepressant prescription; ‘prevalent’ users (those prescribed in the 3 months before pregnancy and into trimester one) only contributed exposed time to the models. Censoring occurred at the earliest of other loss (see Supplementary Information S5), reaching 24 weeks’ gestation, or study end (31 December 2018). Cluster-robust standard errors (clustered by pregnant individual) were employed to account for those who contributed multiple pregnancies to the analysis.

To enhance clinical interpretability, the absolute confounder-adjusted risks (1 minus survival) were estimated using Breslow’s baseline estimator and these were integrated with the hazard ratio (HR) through the G-formula (that estimates the average outcome that would be seen if everyone took antidepressants during trimester one) and bootstrapping for standard errors (1000 repetitions).

The model was run restricted to those with evidence of depression or anxiety in the 12 months before pregnancy and to those with ‘severe’ depression or anxiety, as defined by administered scale standardised scores (like the nine-item Patient Health Questionnaire, Supplementary Information S6) in the 12 months before pregnancy. In addition, receipt of an antidepressant prescription was compared with none in trimester one among those who were prescribed antidepressants in the 3 months before pregnancy.

In an exposure-discordant pregnancy analysis, pregnancies in the same individual were compared. This approach accounts for time-stable confounders, like genetic liability to miscarriage, by design because in a single individual time-stable confounders have the same effect on all pregnancies and therefore are ‘adjusted’ for in the analysis.^36,37^ A stratified Cox model adjusted for the primary adjustment set (except history of miscarriage) was used, where each stratum in the model represented an individual with ≥2 exposure-discordant pregnancies (see Supplementary Information S6).

Propensity-score matching was also performed, following the stepwise process laid out by Desai and Franklin.^38^ The propensity score included both confounders and predictors of the outcome (see Supplementary Table S2)^39^ and was restricted to first pregnancies. The propensity score was estimated using logistic regression, then the final iteration of balancing criteria were applied: exposed and unexposed pregnancies were matched 1:1 without replacement using a caliper of 0.2, and exact matching on number of primary care visits before pregnancy. The Love plot representing the balance achieved by the above criteria can be found in Supplementary Information S6.3 and Supplementary Figures S2–S7.

For the secondary analyses, ‘prevalent’ (≥1 prescription for antidepressants in the 3 months before and during trimester one) and ‘incident’ (≥3 months clear of antidepressant prescriptions before pregnancy; ≥1 prescription during trimester one) antidepressant users were compared with the unexposed group. Analysis was also restricted to those with any depression or anxiety, as well as ‘severe’ illness before pregnancy (see Supplementary Table S3). Individual antidepressant class was compared with the unexposed group. In addition, low, medium, and high doses of antidepressant in trimester one were compared with the unexposed group (see Supplementary Information S3).

In sensitivity analysis, all above analyses were restricted to those with Hospital Episode Statistics data, owing to pregnancy outcome modifications (see Supplementary Information S2). The primary Cox model where exposure was redefined as ≥2 antidepressant prescriptions in trimester one was performed to limit exposure misclassification. The potential for differential pregnancy exclusion, potential bias in the complete records analysis,^40^ and potential bias introduced by competing events (that is, other early pregnancy losses) were investigated.

All analyses were performed in Stata (version 17.0) and R (version 4.3.1).

## Results

**Figure 1.**
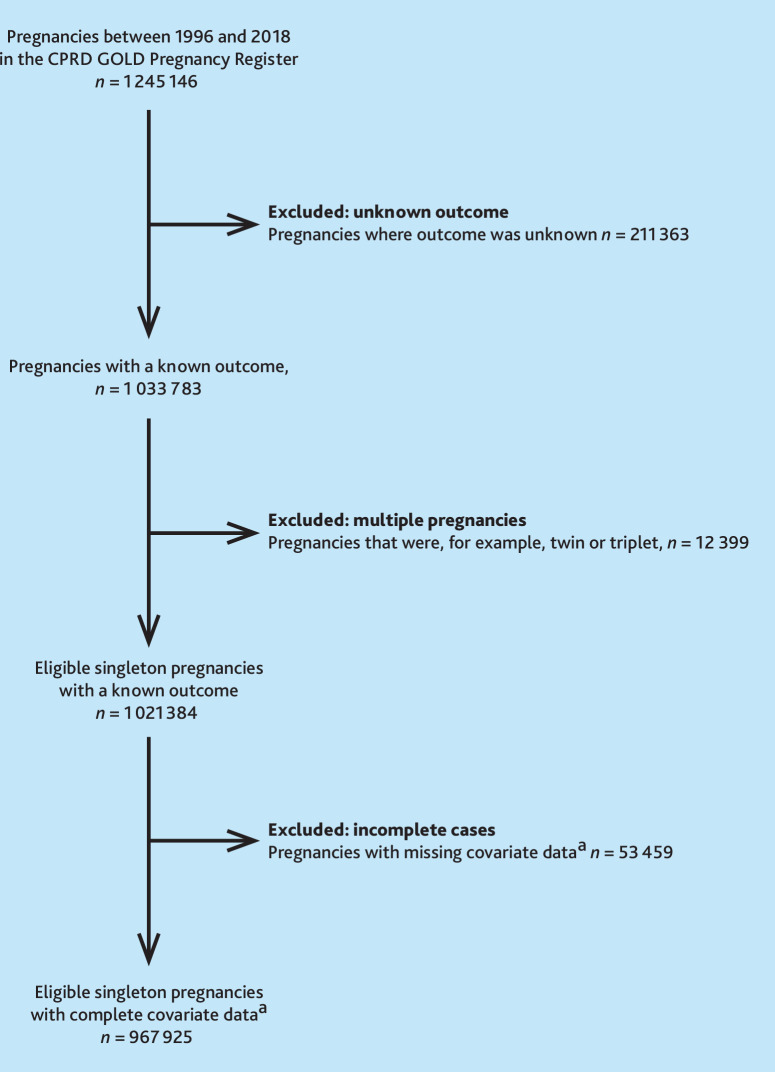
Sample selection and flow of pregnancy episodes through the study. ^a^Complete covariate data refers to the adjustment set in the primary Cox models. Further exclusions were made based on incomplete data in the covariate set used to estimate the propensity score in the propensity-score matched analysis.

The CPRD GOLD Pregnancy Register contained 1 245 146 non-conflicting pregnancies between 1996 and 2018 with sufficient follow-up. Having excluded ‘unknown outcome’ and multiple pregnancies, 967 925/1 245 146 (77.7%, among 661 825 individuals) were eligible and had complete covariate data (Figure 1). Pregnancy outcomes in the eligible sample are summarised in Supplementary Table S4.

During the first trimester, 73 540 were prescribed antidepressants (7.2%). Those prescribed antidepressants during trimester one were slightly older and were more likely to have a BMI ≥30 kg/m^2^ than those who were unexposed. Individuals prescribed antidepressants were more likely to make >10 doctor visits and be using other medications in the year before pregnancy (Table 1).

**Table 1.** Characteristics of pregnant women eligible for inclusion

Characteristic	All (*N* = 1 021 384), *n* (%)	Exposed (*N* = 73 540), *n* (%)	Unexposed (*N* = 947 844), *n* (%)
**Age, years**			
<18	38 690 (3.8)	999 (1.4)	37 691 (4.0)
18–24	232 914 (22.8)	17 613 (24.0)	215 301 (22.7)
25–29	263 042 (25.8)	19 079 (25.9)	243 963 (25.7)
30–34	283 073 (27.7)	19 146 (26.0)	263 927 (27.8)
≥35	203 665 (19.9)	16 703 (22.7)	186 962 (19.7)
**Year of pregnancy**			
1996–2000	119 012 (11.7)	4820 (6.6)	114 192 (12.0)
2001–2005	242 286 (23.7)	14 702 (20.0)	227 584 (24.0)
2006–2010	309 392 (30.3)	20 341 (27.7)	289 051 (30.5)
2011–2015	256 246 (25.1)	22 579 (30.7)	233 667 (24.7)
2016–2018	94 448 (9.2)	11 098 (15.1)	83 350 (8.8)
**Practice-level IMD (quintiles)**			
1 (least deprived)	161 493 (15.8)	9560 (13.0)	151 933 (16.0)
2	165 591 (16.2)	11 025 (15.0)	154 566 (16.3)
3	187 170 (18.3)	13 332 (18.1)	173 838 (18.3)
4	229 209 (22.4)	17 080 (23.2)	212 129 (22.4)
5 (most deprived)	277 921 (27.2)	22 543 (30.7)	255 378 (26.9)
**Ethnicity**			
White	631 614 (61.8)	47 666 (64.8)	583 948 (61.6)
South Asian	31 494 (3.1)	865 (1.2)	30 629 (3.2)
Black	16 706 (1.6)	470 (0.6)	16 236 (1.7)
Other	11 127 (1.1)	324 (0.4)	10 803 (1.1)
Mixed	6589 (0.6)	389 (0.5)	6200 (0.7)
Missing	323 854 (31.7)	23 826 (32.4)	300 028 (31.7)
**Body mass index**			
Underweight (<18.5 kg/m^2^)	33 616 (3.3)	2700 (3.7)	30 916 (3.3)
Healthy weight (18.5–24.9kg/m^2^)	465 110 (45.5)	28 847 (39.2)	436 263 (46.0)
Overweight (25.0–29.9 kg/m^2^)	238 249 (23.3)	17 403 (23.7)	220 846 (23.3)
Obese (≥30.0 kg/m^2^)	179 700 (17.6)	18 540 (25.2)	161 160 (17.0)
Missing	104 709 (10.3)	6050 (8.2)	98 659 (10.4)
**Previous miscarriage**			
Yes	160 994 (15.8)	14 413 (19.6)	146 581 (15.5)
**Parity**			
0	485 775 (47.6)	27 226 (37.0)	458 549 (48.4)
1	346 248 (33.9)	24 698 (33.6)	321 550 (33.9)
2	131 356 (12.9)	13 818 (18.8)	117 538 (12.4)
≥3	58 005 (5.7)	7798 (10.6)	50 207 (5.3)
**Mental health history^a^**			
Depression	252 356 (24.7)	56 305 (76.6)	196 051 (20.7)
Anxiety	154 394 (15.1)	33 841 (46.0)	120 553 (12.7)
Severe mental illness^b^	5079 (0.5)	1774 (2.4)	3305 (0.3)
**Primary care visits in the 12 months before pregnancy**			
0	119 052 (11.7)	4976 (6.8)	114 076 (12.0)
1–3	262 141 (25.7)	4665 (6.3)	257 476 (27.2)
4–10	419 751 (41.1)	24 411 (33.2)	395 340 (41.7)
>10	220 440 (21.6)	39 488 (53.7)	180 952 (19.1)
**Smoking status around the start of pregnancy**			
Non-smoker	414 763 (40.6)	19 925 (27.1)	394 838 (41.7)
Current smoker	304 897 (29.9)	32 128 (43.7)	272 769 (28.8)
Ex-smoker	248 265 (24.3)	19 407 (26.4)	228 858 (24.1)
Missing	53 459 (5.2)	2080 (2.8)	51 379 (5.4)
**Other prescriptions 12 months before pregnancy**			
Antipsychotics	865 (0.1)	497 (0.7)	368 (0.04)
Mood stabilisers	9912 (1.0)	3001 (4.1)	6911 (0.7)
Folic acid (5 mg)	58 830 (5.8)	7172 (9.8)	51 658 (5.5)

ab Identified using Read and International Classification of Diseases, 10th Revision codes from primary care data and Hospital Episode Statistics data (for patients who had linked secondary care data), respectively. Bipolar disorder, psychosis, or schizophrenia. IMD = Index of Multiple Deprivation.

Those excluded because of an ‘unknown outcome’ pregnancy were characteristically comparable with included individuals, other than there being more missing data and more doctor visits before pregnancy (see Supplementary Table S5).

### Primary analyses

**Figure 2.**
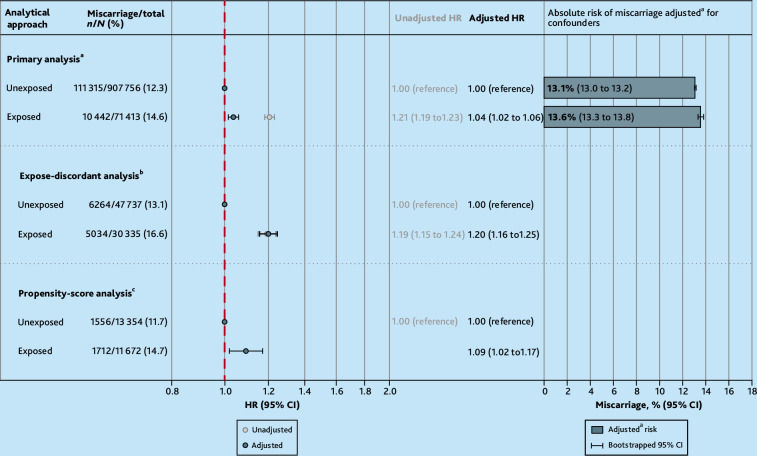
Adjusted relative and absolute risks from the primary analyses. ^a^Adjusted for maternal age; pregnancy year; practice-level Index of Multiple Deprivation quintile; history of miscarriage; smoking status around the start of pregnancy; parity at the start of pregnancy; use of high-dose folic acid, antipsychotics, or antiseizure medication in the 12 months before pregnancy; number of primary care consultations in the 12 months before pregnancy; and severe mental illness, depression, or anxiety ever before the start of pregnancy. ^b^Primary analysis adjustment set with history of miscarriage dropped. ^c^Primary adjustment set additionally included in the propensity score: area of residence; alcohol use around the start of pregnancy; illicit drug use in the year before pregnancy; diabetes; endometriosis; polycystic ovary syndrome; teratogen use in the year before pregnancy; and other potential antidepressant indications ever before pregnancy. HR = hazard ratio.

Among pregnancies with complete covariates, antidepressant use during first trimester was associated with miscarriage in the unadjusted models (HR 1.21, 95% CI = 1.19 to 1.23). On adjustment, the effect lessened (adjusted HR [aHR] 1.04, 95% CI = 1.02 to 1.06), with a standardised miscarriage risk of 13.6% (95% CI = 13.3 to 13.8) among those prescribed and 13.1% (95% CI = 13.0 to 13.2) among those not prescribed antidepressants (Figure 2 and Supplementary Table S6). This finding was consistent when exposure was defined as ≥2 antidepressant prescriptions in trimester one (see Supplementary Table S7), when restricting to those with depression or anxiety noted, and when restricting to those with ‘severe’ illness noted in the year before pregnancy (see Supplementary Table S8). Among those prescribed antidepressants in the 3 months before pregnancy, the risk of miscarriage was no different between those who continued the antidepressants into trimester one and those who discontinued them before pregnancy (see Supplementary Table S9).

When comparing exposure-discordant pregnancies within the same birthing parent, thereby accounting for all unobserved time-stable (such as, genetics) and observed confounders, the adjusted estimate was analogous to the unadjusted primary Cox model (aHR 1.20, 95% CI = 1.16 to 1.25) (Figure 2).

**Figure 3.**
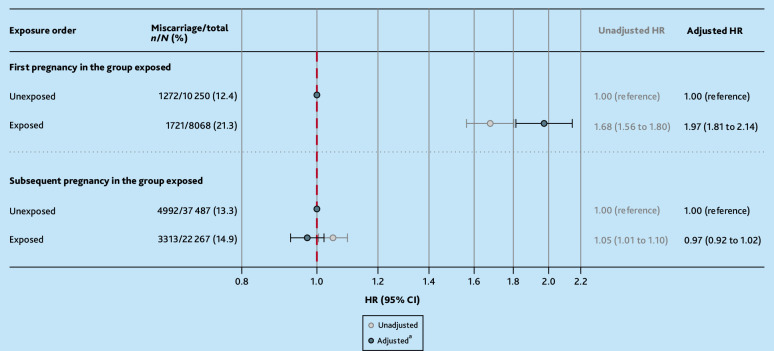
Exposure-discordant pregnancy sensitivity analysis, restricting first to exposure-discordant groups where the first pregnancy was antidepressant exposed and subsequent pregnancies in the group were not, then to groups where subsequent pregnancies were exposed to antidepressants but first pregnancies in the group were not. ^a^Primary analysis adjustment set (maternal age; pregnancy year; practice-level Index of Multiple Depriviation quintile; smoking status around the start of pregnancy; parity at the start of pregnancy; use of high-dose folic acid, antipsychotics, or antiseizure medication in the 12 months before pregnancy; number of primary care consultations in the 12 months before pregnancy; and severe mental illness, depression, or anxiety ever before the start of pregnancy) with history of miscarriage dropped. HR = hazard ratio.

The risk of miscarriage when the first pregnancy in the exposure-discordant group of pregnancies was exposed and then when a subsequent pregnancy in the group was exposed was further investigated. This revealed that first trimester antidepressant use was only associated with miscarriage when the first pregnancy in the group was exposed (aHR 1.97, 95% CI = 1.81 to 2.14), not when a subsequent pregnancy was exposed (aHR 0.97, 95% CI = 0.92 to 1.02) (Figure 3 and Supplementary Table S10), highlighting the potential importance of pregnancy order or non-shared, time-varying confounding in these analyses as opposed to antidepressants.

When matching pregnancies on propensity score, the current findings were consistent with those from the primary Cox model (aHR 1.09, 95% CI = 1.02 to 1.17) (Figure 2).

### Secondary analyses

**Figure 4.**
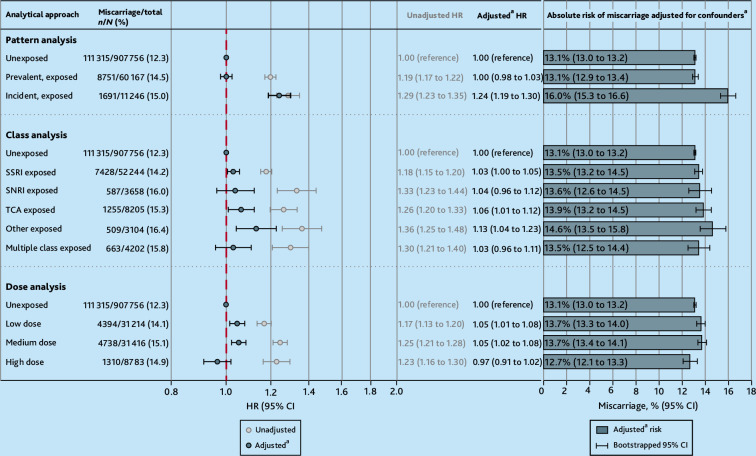
Adjusted relative and absolute risks from the secondary analyses. ^a^Adjusted for maternal age; pregnancy year; practice-level Index of Multiple Depriviation quintile; history of miscarriage; smoking status around the start of pregnancy; parity at the start of pregnancy; use of high-dose folic acid, antipsychotics, or antiseizure medication in the 12 months before pregnancy; number of primary care consultations in the 12 months before pregnancy; and severe mental illness, depression, or anxiety ever before the start of pregnancy. HR = hazard ratio. SNRI = serotonin noradrenaline reuptake inhibitor. SSRI = selective serotonin reuptake inhibitor. TCA = tricyclic antidepressant.

In unadjusted models, both ‘prevalent’ and ‘incident’ use was associated with an increased hazard of miscarriage, but adjustment for covariates only attenuated ‘prevalent’ use and ‘incident’ use remained above 1 (aHR 1.00, 95% CI = 0.98 to 1.03 and aHR 1.24, 95% CI = 1.19 to 1.30, respectively), despite similarity across their measured characteristics (see Supplementary Table S11). After adjustment, the estimated absolute risk of miscarriage adjusted for confounders was the same for unexposed pregnancies and ‘prevalent’ use (13.1%, Figure 4). The estimates did not change when restricting it to those with ≥2 first trimester prescriptions or among an indication-based sample (see Supplementary Table S6 and S7).

Selective serotonin reuptake inhibitor (SSRI), serotonin noradrenaline reuptake inhibitor, tricyclic, and ‘other’ antidepressant use during trimester one were associated with a slight increase in risk of miscarriage versus no use. Low and medium dose were associated with miscarriage, where high dose attenuated to the null compared with those who were unexposed, following adjustment for covariates (Figure 4).

### Sensitivity analyses

The results were consistent when restricting each analysis to those with linked data (see Supplementary Table S12). Having depression noted in the 12 months before pregnancy was modestly associated with having an ‘unknown outcome’ pregnancy (see Supplementary Table S13). When adding ethnicity and BMI to the adjustment set, the estimates did not change (see Supplementary Table S14). When assessing potential bias in the complete records analysis,^40^ those who had a miscarriage were more likely to have missing data in covariates (see Supplementary Table S15). Among individuals who were nulliparous, the estimates did not change substantially (see Supplementary Table S16). When including ectopic and molar pregnancies in the definition of miscarriage, the results were consistent with the primary analysis (see Supplementary Table S17).

## Discussion

### Summary

This population-based cohort study of nearly 1 million pregnancies in the UK found little evidence that first trimester antidepressant use substantially increases the risk of miscarriage, with no evidence suggesting that pre-pregnancy antidepressant use into trimester one increases the risk of miscarriage. The conclusions are less clear for women initiating antidepressants in the first trimester; however, issues including gestational dating in early pregnancy and probable residual confounding prohibit the authors from interpreting this observation as causal. The small observed increases in absolute risk, even if causal, are clinically insignificant (13.1% versus 13.6%) and will provide a useful clinical decision-making aid.

### Strengths and limitations

This study is large, with >600 000 individuals from a UK-representative sample,^32^ contributing nearly 1 million pregnancies, improving estimate precision. It leverages multiple methods and comparators to explore the role of confounding by indication and data issues encountered when performing pharmacoepidemiologic studies using observational data. The use of the CPRD GOLD Pregnancy Register made it possible to build on the systematic approach taken by Minassian *et al.* ^33^ The use of cause-specific time-to-event models made it possible to retain pregnancies at risk of miscarriage while ongoing that neither ended in the outcome nor reached the end of follow-up, in other words the pregnancy ended in another type of loss. Thus, the denominator was not differentially deflated by exposure status; had other losses been omitted from the analysis, the proportion of pregnancies that ended in miscarriage would have been artificially inflated among the exposed group more so than the unexposed group.

The application of eligibility criteria inevitably led to a smaller and more select sample than the full CPRD GOLD population. Confounding is likely still present in these analyses. Although the study adjusted for depression and anxiety in the main analysis and restricted it to those with recent or ‘severe’ depression and/or anxiety in the 12 months before pregnancy, residual confounding by underlying severity of indication for treatment surely contributed to the results observed, particularly for ‘incident’ use, given the inability to account for unmeasured factors in spite of the extensive analyses with observed data. Regardless, confounding by indication was targeted carefully and robustly in the current study while taking a considered approach to the risk-of-bias amplification in an indication-based sample by not solely relying on this approach for indication adjustment.^41^

Differential exposure misclassification was a concern as miscarriages were more likely to have an imputed gestational length than deliveries;^33^ imputed gestational length may have resulted in more miscarriages being misclassified as prescribed antidepressants than deliveries.^42^ The possibility for reverse causation may explain some of the miscarriages observed in the ‘incident’ group, where antidepressants were sought following a miscarriage; another potential consequence of potentially higher misclassification burden among the exposed group. Those seeking health care for depression, anxiety, or other indications treated with antidepressants may be more likely to report pregnancies and early losses than those not engaging with health care for other reasons.

Given that the presence of these limitations cannot be easily quantified, it is reassuring that the primary results would translate to a modest increase in absolute risk from 13.1% among those unexposed to 13.6% among those prescribed antidepressants (that is, a number needed to harm of 200) if causal.

### Comparison with existing literature

A systematic review and meta-analysis of 29 studies by Smith *et al* showed a slight increased risk of miscarriage following antidepressant use during pregnancy and noted several methodological weaknesses in the previous literature.^18^ A large Danish study found an association between SSRI use during pregnancy and trimester one miscarriage of a similar magnitude to the present findings.^6^ They concluded that confounding by lifestyle factors and indication were responsible for the association, given that they observed a complete attenuation when compared with individuals with unmedicated depression during pregnancy. Another study highlighted the challenges faced in the field, particularly when dealing with confounding by indication.^43^

Some studies comparing antidepressant use with unmedicated depression have found a complete attenuation,^6,43^ whereas others have found a persistent risk of miscarriage following antidepressant use.^5,44–46^ Few studies have included indication-based covariates in a multivariable model,^43,47,48^ which may be preferable over an indication-based sample.^41^ Interestingly, the unadjusted estimate from the current study (HR 1.21, 95% CI = 1.19 to 1.23) is similar to the summary estimate observed in Smith and colleague’s review (1.24, 95% CI = 1.18 to 1.31).^18^

The exposure-discordant pregnancy analysis showed that the risk of miscarriage was higher in an exposed pregnancy compared with an unexposed one in the same individual. When investigating this further by looking at pregnancy order, the current study showed that the risk was only observed when the first pregnancy was exposed compared with subsequent unexposed pregnancies. This sensitivity analysis speaks to the unpredictability of exposure-discordant analyses when there is potentially time-varying confounding and carryover effects at play; these biases may amplify estimates from discordant analysis in many situations.^37,49^

The current study shows that ‘incident’ use of antidepressants during trimester one was associated with a higher risk of miscarriage compared with no use. Differences in risk for ‘incident’ but not ‘prevalent’ antidepressant use have been observed previously for some neurodevelopmental outcomes.^50^ The introduction of a new drug substance into the body could disrupt early fetal development, but there are several other plausible explanations. Residual confounding by severity of indication,^51^^,^^52^ health-seeking behaviour, or data artefacts, such as the imputation of pregnancy length, might be partially driving the association.

### Implications for practice

The findings are reassuring for prescribing clinicians and individuals concerned about antidepressant use during early pregnancy and miscarriage, providing both parties with robust supportive evidence for informed decision making in clinic. In a recent qualitative study, women cited concerns about the possibility of pregnancy loss and fear of doing something that would compromise their pregnancy, that is, take antidepressants.^53^ The current study found that antidepressant use during trimester one does not substantially increase the risk of miscarriage, with no evidence suggesting taking antidepressants before pregnancy and into trimester one increases the risk of miscarriage. Despite observing an elevated risk for ‘incident’ users, the overall relative risk translated to a modest increase in absolute risk and other biases cannot be ruled out.

## Data

Access to CPRD data, including UK primary care data and linked data such as Hospital Episode Statistics, is subject to protocol approval as per CPRD’s guidelines. Authors are unable to share the data in its raw form; however, all analytical code and codelists are open-source and found via the following links: https://github.com/flozoemartin/Miscarriage and https://github.com/flozoemartin/codelists.
